# Efficacy and Safety of A Liposome-Based Vaccine against Protein Tau, Assessed in Tau.P301L Mice That Model Tauopathy

**DOI:** 10.1371/journal.pone.0072301

**Published:** 2013-08-19

**Authors:** Clara Theunis, Natalia Crespo-Biel, Valérie Gafner, Maria Pihlgren, María Pilar López-Deber, Pedro Reis, David T. Hickman, Oskar Adolfsson, Nathalie Chuard, Dorin Mlaki Ndao, Peter Borghgraef, Herman Devijver, Fred Van Leuven, Andrea Pfeifer, Andreas Muhs

**Affiliations:** 1 Experimental Genetics Group, Department Human Genetics, KU Leuven, Leuven, Belgium; 2 ACImmune, Lausanne, Switzerland; Thomas Jefferson University, United States of America

## Abstract

Progressive aggregation of protein Tau into oligomers and fibrils correlates with cognitive decline and synaptic dysfunction, leading to neurodegeneration in vulnerable brain regions in Alzheimer's disease. The unmet need of effective therapy for Alzheimer's disease, combined with problematic pharmacological approaches, led the field to explore immunotherapy, first against amyloid peptides and recently against protein Tau. Here we adapted the liposome-based amyloid vaccine that proved safe and efficacious, and incorporated a synthetic phosphorylated peptide to mimic the important phospho-epitope of protein Tau at residues pS396/pS404. We demonstrate that the liposome-based vaccine elicited, rapidly and robustly, specific antisera in wild-type mice and in Tau.P301L mice. Long-term vaccination proved to be safe, because it improved the clinical condition and reduced indices of tauopathy in the brain of the Tau.P301L mice, while no signs of neuro-inflammation or other adverse neurological effects were observed. The data corroborate the hypothesis that liposomes carrying phosphorylated peptides of protein Tau have considerable potential as safe and effective treatment against tauopathies, including Alzheimer's disease.

## Introduction

Microtubule-associated protein Tau (MAPT) is a soluble, naturally unfolded protein that is normally bound to microtubuli, spacing them apart to support microtubuli-mediated axonal transport [1]–[4]. In adult brain, the binding of protein Tau to microtubuli is controlled by dynamic phosphorylation. Under physiological conditions each Tau molecule carries a limited number of phosphate groups [5] and the protein remains soluble despite its unfolded state. Under pathological conditions, Tau becomes phosphorylated at many more sites, which is proposed to compromise its normal functions. A major analytical problem is posed by the high number of potential sites that, in the unfolded state of protein Tau, can be targeted by a wide range of kinases, producing virtually thousands of phospho-Tau isomers.

In all tauopathies, increased phosphorylation and aggregation of protein Tau is evident, although it remains disputed what is cause, consequence and correlation. The favored hypothesis maintains that in first instance a trigger, e.g. a mutation in FTDP-17 or a co-morbid event such as increased levels of amyloid peptides in Alzheimer's disease (AD), activates kinases and/or inactivates phosphatases. The resulting increased phosphorylation of protein Tau eventually releases it from the axonal microtubuli. Unbound protein Tau can become dislocated from the axons into the soma and dendrites, where it is prone to further phosphorylation. The progressive phosphorylation of protein Tau is accompanied by aggregation, first into oligomers and subsequently into the larger fibrils that typically litter the soma and processes as neurofibrillary tangles (NFT) and neuropil threads. In AD brain, the latter make up 80% of the tauopathy that is typically detected as argyrophilic deposits in post-mortem brain sections [6]–[9].

Alzheimer's disease is classically described and diagnosed by the unique combination of amyloid and Tau pathology. Recent findings revealed the closer correlation of cognitive decline with Tau-related brain defects than with amyloid load which can even be high in cognitively normal individuals [7], [9]–[11]. Additionally, genetic defects and mutations in exons and introns of the gene coding for protein Tau on chromosome 17 (*MAPT*) were directly linked to a subtype of familial frontotemporal dementia. This demonstrates that tauopathy alone can be neurotoxic, even in the absence of amyloid or other aggregated proteins [12]–[15]. The combined data promote protein Tau to a prime scientific and therapeutic target in AD.

At present, no effective treatment is available that prevents, halts or reverses amyloid or Tau pathology. Drugs currently prescribed to demented patients act symptomatically, without modifying the still largely unknown molecular causes of AD. The search for therapeutic interventions that counteract the pathological effects of amyloid peptides and of protein Tau is at present being intensely pursued in different directions [16]–[18]. Immunotherapy has gradually emerged as a promising approach against protein misfolding diseases that bring about neurodegeneration. Vaccination was and still is aimed on amyloid peptides in more than 30 ongoing clinical studies, mostly relying on passive vaccination (www.clinicaltrials.gov). Many clinical trials have been abrogated for lack of efficacy, and for safety reasons [19], [20]. The threat of auto-immune reactions, inherent to active immunotherapy with a human peptide, has emphasized the need for early and more elaborate preclinical assessment of safety prior to clinical trials.

Here, we report the adaptation of the validated liposome-based vaccine technology [21]–[24]. We elected to target pathological protein conformers of protein Tau by incorporation of short phosphorylated peptides into liposomes to mimic pathological Tau epitopes. The epitope selected for this novel approach of Tau directed immunotherapy corresponds to the well-known series of serine residues S396/S400/S404 that become differentially phosphorylated, particularly in pathology, by GSK3 kinases [25]. The epitopes of several monoclonal antibodies that define late stages in Tau pathology, e.g. AD2 and PHF1 among others, are located in this region [25]–[29]. Specifically, the liposomal vaccine ACI-35 contained a 16-mer synthetic peptide corresponding to human protein Tau sequence 393–408, with phosphorylated residues S396 and S404. The ability of liposome-based vaccines to induce robust and specific antibody responses was assessed in wild-type and in transgenic mice that model tauopathy [30], [31]. Furthermore, we extensively evaluated the safety of the vaccine to show that the benefits in clinical condition and improved survival were evident without any signs of neuroinflammation or neurological side-effects.

## Materials and Methods

### Ethics statement

All animal experiments were performed by certified researchers conform the regional, national and European regulations concerning animal welfare and animal experimentation, authorized and supervised by the university animal welfare commission (Ethische Commissie Dierenwelzijn, KULeuven). We formally declare that we comply to the European FP7-Decision 1982/2006/EC, Article 611, i.e. all research activities is carried out in compliance with fundamental ethical principles and all experiments are approved and overlooked by the respective Animal Welfare Commissions.

### Vaccine

The preparation of the liposomal vaccine was based on a previously described protocol [23]. For the long term study in wild-type mice, the lipids di-myristoylphosphatidylcholine (DMPC), di-myristoylphosphatidylglycerol (DMPG), cholesterol, the adjuvant monophosphoryl lipid A (MPLA) (Avanti Polar Lipids) and the synthetic Tau-derived tetrapalmitoylated phosphopeptides were dissolved at molar ratios of 9∶1∶7∶0.2∶0.1 in chloroform. Following mixing at RT, the solution was evaporated under reduced pressure at 40°C, and then under high vacuum for 1 hour. The resulting thin-film was rehydrated in PBS by mixing at RT for 18 hours, and the resulting liposomes were stored at 5°C. The liposomal vaccine for subsequent studies was generated according a modified protocol. DMPC, DMPG, cholesterol and MPLA were dissolved at molar ratios 9∶1∶7∶0.05 in ethanol at 60°C. The solution was diluted with PBS pH 7.4 and concentrated by ultrafiltration. The liposomes were homogenized and sized by sequential extrusion through polycarbonate filters with pores 0.2 *µ*m (EmulsiFlex-C5; Avestin, Canada). The resulting liposomes were diluted in PBS pH 7.4 and heated to 60°C for addition of the tetrapalmitoylated phosphopeptide, dissolved in PBS (pH 11.5) with 2.0% β-octylglucoside. After mixing at 60°C for 30 min, the vaccines were concentrated by ultrafiltration and diafiltration against PBS (pH 7.4). The resulting liposomes were sterilized by ultrafiltration through 0.2 *µ*m polycarbonate syringe filters and stored at 5°C. Specifically, we generated, analyzed and tested liposomal vaccines that carried peptides corresponding to residues 393–408 in protein Tau (numbering of the Tau441 isoform) with S396 and S404 phosphorylated. These were designated ACI-35-Tau393-408[pS396/pS404] liposomes, denoted as ACI-35.

### Circular dichroism (CD) spectroscopy of ACI-35 Tau393-408[pS396/pS404] liposomes

ACI-35 liposomes were diluted in PBS to yield final peptide concentrations of 9.5 *µ*M. Liposomes with identical composition but lacking peptides were used as negative controls for baseline subtraction. CD spectra were recorded from samples in quartz cuvettes (0.1 cm path length) at 23°C (Jasco-815 spectropolarimeter) at 195–250 nm, with 1.0 nm bandwidth and 0.5 nm resolution, at scan speed of 50 nm/min with response time of 1 sec. Blank spectra from 8 scans were averaged and subtracted from the averaged 8 scans of each sample. The obtained spectrum ([θ]_obs_, degrees) was smoothed after conversion to mean residue molar ellipticity ([θ], degrees cm^2^ dmol^−1^) by the equation [θ] = [θ]_obs_.(MRW/10.l.c), with MRW as the mean residue molecular weight (MW/number of residues), l optical path length (cm) and c concentration (g/cm^3^).

### Mice

The ACI-35 liposomal vaccine was tested for the induction of specific, safe and long-lasting immune responses in wild type C57BL/6 and in transgenic Tau.P301L mice, our validated model for tauopathy [12], [30]–[38]. Tau.P301L mice express the longest human Tau441 isoform with the Tau.P301L mutation under control of the mouse Thy1 gene promoter in the FVB/N genetic background [30]. Ageing Tau.P301L mice develop a moribund tauopathy with typical formation of Tau aggregates as neurofibrillary tangles in somata and neuropil threads in dendrites, first in hindbrain starting at age 6 months and then progressing to the forebrain at age 9 months. Precocious death sets in around age 7–8 months (range 7–12 months, mean age 9.5 months) with practically no survivors beyond age 12 months [37]. The terminal stage of Tau.P301L mice is mainly caused by brainstem Tau pathology and evolves rather rapidly, taking no more than 2 to 3 weeks after the first severe signs of motor impairment become apparent by clasping and rotarod testing. Terminal Tau.P301L mice lose body weight, display a dystonic posture with progressive paralysis of hind- and forelimbs, and their breathing problems cause exhaustion and asphyxia, resulting in death [30]–[37].

### Clinical condition

The general condition of all Tau.P301L mice was monitored weekly and clasping score and body weight were recorded. Clasping, defined as the retraction of legs towards the body when suspended by the tail, was scored from 0 to 4, according to the number of legs involved. Tau.P301L mice are defined as ‘terminal’ when clasping score is 4 and bodyweight drops below 16 grams for females. Terminal Tau.P301L mice are represented in the results section with “score 5” and were sacrificed for ethical reasons.

Motor coordination and balance was assessed in a standard automated rotarod apparatus, comparing 5 mice simultaneously on the rotating rod (diameter 3 cm; Rotarod, Sandown Scientific, UK). Prior to treatment, all mice were habituated to the apparatus during 3 training sessions on 3 consecutive days, sufficient to establish a baseline level of performance. Each training session comprised 3 runs of 3 min at 20 rpm with 1 hour rest in between runs. A soft foam cushion was placed beneath the rods, and mice that fell during the training session were placed back onto the rod to complete the session. During treatment, control and test mice were compared biweekly in blinded sessions, with the rotating rod accelerating from 4 to 40 rpm over 5 min. The time that each mouse remained on the revolving rod was recorded automatically (maximum 300 sec).

### Vaccination

Adult C57BL/6 female mice of age 6 months (n = 10) were immunized by subcutaneous administration of 200 *µ*l liposomal vaccine. After three injections with two week intervals, the mice were bled, and eventually vaccinated again after a recess of 3 months to define the stability in time and the memory of the immune response. Similarly, adult female Tau.P301L mice (n = 60 for the ACI-35 vaccine; n = 57 for placebo) were vaccinated beginning at age 6 months, when they are still pre-symptomatic for motor defects but with the first signs of tauopathy appearing in their brainstem. Surviving mice were sacrificed at age 9 months. Over the observation period of 3 months, the Tau.P301L mice received 5 subcutaneous injections of 200 µl of the ACI-35 vaccine. The first three doses were administered with two week intervals (d0, d14, d28), followed by 2 monthly injections (d56, d84). Blood was collected at the indicated time-points, both before (denoted B0) and during vaccination (denoted B1 to B3), via the tail vein during life and at sacrifice by heart puncture (denoted HP).

The vaccinated Tau.P301L mice were sacrificed either at age 9 months or earlier when their remaining life-span was judged to be shorter than 24 hours, termed terminal phase, described above. For euthanasia, mice were anesthetized (Nembutal; 120 mg/kg i.p.) and blood was collected by heart puncture, followed by transcardiac perfusion with ice-cold saline (2 ml/min, 2 min). The brain was removed and the right hemisphere was dissected (cerebrum, brainstem, cerebellum) and the tissues snap-frozen for biochemical analysis. The left hemisphere was fixed for immunohistochemistry in 4% paraformaldehyde in PBS (4°C, 18 hours) and stored (0.1% sodium azide, PBS, 4°C) until cut for free-floating vibratome sections (40 µm).

### Serology

Serum IgG titers were determined by ELISA on plates coated with the phosphorylated Tau393-408 [pS396/pS404] peptide (pTau peptide) or the non-phosphorylated Tau393-408 peptide as negative control (Tau peptide). In parallel, antisera were analyzed by specific ELISA for reaction on full length recombinant human protein Tau, either not phosphorylated as produced in bacteria (Tau protein) (SignalChem, Canada) or phosphorylated as produced in yeast (pTau protein) [39], [40]. The 96-well plates were coated with peptides (10 µg/ml) or proteins (1 µg/ml) by overnight incubation at 4°C. After washing (0.05% Tween20 in PBS) non-specific binding sites were blocked with 1% bovine serum albumin (BSA) in the same buffer. Subsequently, serial dilutions of mouse antisera were incubated for 2 hours at 37°C and after extensive washing, the immune complexes were quantified by reaction with secondary anti-mouse IgG antibody conjugated to alkaline phosphatase (Jackson Labs, USA) for 2 hours at 37°C. After incubation with para-nitrophenylphosphate for 1 hour at RT in the dark, the reaction was stopped and the optical density was recorded at 405 nm.

### Immunohistochemistry

We performed Tau-Pathology Immuno-Reaction (TAUPIR) with anti-sera from immunized mice to define their specificity on free-floating vibratome sections (40 µm) from old bigenic GSK3βxTauP301L mice (biGT). The biGT mice develop dramatic tauopathy with tangles and neuropil threads in forebrain [31]. After endogenous peroxidase activity was eliminated by incubating brain sections in 1.5% H_2_O_2_ in PBS:MeOH (1∶1) (15 min, RT), sections were washed with 0.1% TritonX100 in PBS (PBST) and incubated (30 min, RT) in blocking buffer (10% fetal calf serum in PBST). Antiserum was diluted, as indicated in the results section and figure legends, for overnight incubation at 4°C. After washing, sections were incubated (1 hr, RT) with goat anti-mouse IgG antibody conjugated with horseradish peroxidase (DAKO, Denmark) diluted 1/500 in blocking buffer. Subsequently, sections were washed 3 times with PBS and incubated in 50 mM Tris/HCl pH 7.6 for 5 min, before detection of immune complexes by incubation with diaminobenzidine and H_2_O_2_ in 50 mM Tris.HCl (pH 7.6). The reaction was stopped by washing the sections 3 times in PBS after which they were transferred to silanized glass slides and air-dried (50°C, 2 hrs). Counterstaining was performed by incubation in Mayers hematoxylin (Fluka, Buchs, Switzerland) for 1 min and stopped by washing with tap-water (4 min). Sections were dehydrated in graded ethanol series, immersed in xylol (2×1 min) and mounted (DePeX; BDH, Poole, UK) under glass cover-slips for analysis by light microscopy and dedicated software (IM500, Leica, Belgium).

To detect and to quantify specific phospho-Tau epitopes in brain sections of vaccinated and control Tau.P301L mice, IHC was performed by similar TAUPIR using AT100 (anti-pS212 and pS214 Tau; Innogenetics, Belgium) and pS422 (anti-pS422 Tau; Invitrogen, UK) as primary antibodies and the respective secondary anti-mouse or anti-rabbit IgG. Inflammation was analyzed with specific primary antibodies against GFAP (DAKO, Denmark), CD45 and MHCII (BD Pharmingen, UK). The corresponding secondary antibodies against rabbit or rat IgG were derived in goats and conjugated to biotin (Vector,UK), where after the signal was amplified by incubating the sections for 30 min in ABC-complex (Vector, UK).

### Biochemical analysis: tissue fractionation and western blotting

Frozen brain was homogenized with a motor-driven glass-pestle homogenizer (700 rpm, in ice-bath) in 6 volumes of cold homogenization buffer: 25 mM Tris.HCl (pH 7.6), 150 mM NaCl, 1 mM EDTA, 1 mM EGTA, 30 mM NaF, 0.2 mM Na_3_VO_4_, 1 nM okadaic acid, 1 mM phenylmethylsulfonylfluoride (PMSF), 5 mM Na_4_P_2_O_7_, complete protease inhibitor cocktail (1 tablet CPIC per 12 ml; Roche, Brussels, Belgium). The total homogenate (TH) was centrifuged (30 min, 150000×g, 4°C) and the resulting supernatant denoted soluble S1 fraction was stored at −20°C until analysis. The pellet was solubilized in 200 µl or 400 µl, respectively for brainstem and cerebrum, cold 10 mM Tris.HCl (pH 7.4), 0.8 NaCl, 10% sucrose, 1 mM EGTA, 1 mM PMSF, and centrifuged (10 min, 14000×g, 4°C). The resulting supernatant was adjusted to 1% sarcosyl, and after incubation for 1 hr at RT, centrifuged (30 min, 150000×g, 4°C) to yield supernatant (SST, sarcosyl soluble Tau) and sarcosyl insoluble Tau (SInT) as the pellet. SInT fractions were resuspended in 50 µl TBS (10 mM Tris, pH 7.4, 0.8 M NaCl) and stored at −20°C until analysis.

Fraction TH, S1 and SInT were diluted in TBS (respectively 1∶5, 1∶5 and 1∶2) and mixed with an equal volume of 2× SDS-PAGE sample buffer: 125 mM Tris/HCl (pH 6.8), 4% (w/v) sodium dodecyl sulfate (SDS), 20% glycerol, 0.01% bromophenol blue, 5% (v/v) beta-mercaptoethanol. Samples were heated at 95°C for 10 min, and centrifugation to cool and collect condensed water (14000 rpm, 10 sec). Proteins were separated in 10% Tris-glycine SDS-PAGE gels (Anamed, Germany) in electrophoresis buffer (25 mM Tris (pH 8.6), 192 mM glycine, 0.1% SDS) and transferred to nitrocellulose membrane (Hybond-ECL; GE Healthcare, Brussels, Belgium) in transfer buffer (25 mM Tris.HCl, pH 8.6, 190 mM Glycine, 20% methanol). Proper protein loading and separation was routinely monitored by staining the membranes with 0.1% ponceau red in 5% acetic acid. Non-specific binding to membranes was blocked by incubation in 5% non-fat dry milk in TBST (0.1% Tween-20, 50 mM Tris.HCl, pH 7.6, 150 mM NaCl) prior to overnight incubation at 4°C with primary antibody as indicated: Tau5 (anti-pan Tau; BD bioscience, USA), HT7 (anti-human Tau; Thermo Scientific, USA), pS396 (Invitrogen, UK), AT180 (anti-pT231, Innogenetics, Belgium), pS404 (Sigma, USA) and GFAP (DAKO, Denmark) all diluted in the same blocking buffer. After washing in TBST, blots were incubated for 1 hour at RT with secondary goat anti-mouse or anti-rabbit, HRP-conjugated (DAKO, Denmark) diluted 1/10 000 in blocking buffer. Following another wash with TBST, blots were incubated for detection by chemiluminescence and quantitative analysis by dedicated reagents, instruments and software (ECL; LAS4000; Image Quant; GE Healthcare, Belgium).

### ELISPOT study

The Tau/Alum/CpG vaccine was prepared by the following protocol. Full-length lyophilized Tau protein (Signal Chem, Canada) was resuspended in PBS at a concentration of 1 mg/ml and filtered (0.22 µM). Alum (2% Al2O3 corresponding to 3% Al(OH)3 Alhydrogel, Brenntag Biosector, Denmark) was added to obtain a final concentration of 0.25 mg/ml Tau to 5 mg/ml Al(OH)3. CpG ODN 1668 (Microsynth, Switzerland) at 10 nm was mixed to the Tau/Alum solution to get a final concentration of 50 nmol/ml (300 µg/ml). The final ratio of Tau/Alum/CpG is 50/1000/60 µg/dose.

C57BL/6 mice of 11 weeks (n = 10) received s.c. injections of ACI-35 or the Tau/Alum/CpG vaccine on day 0 (d0) and day 10 (d10). Plasma was collected 3 days (d-3) before the first immunization and at day 15 (d15) and stored at −80°C until use. Mice were sacrificed 16 days after the first immunization and the spleens were taken for analysis of T cell responses.

Cytokine production of pTau/Tau peptides or Tau protein-specific T cells was assessed by ELISPOT as previously described [24]. Single cell suspensions were prepared from spleens of immunized mice or from naive mice as negative controls. The cells were serially diluted and incubated at 37°C for 48 hours in precoated 96-well plates (MABTECH, Sweden) with either concanavalin A (Con A: 5 µg/ml, GE Healthcare, UK), pTau or Tau peptides (0.5 or 0.6 µM) or recombinant protein Tau (2.1 µM). The cells were washed and incubated 2 hours at 37°C with biotinylated anti-mouse IFNγ and anti-IL-4 monoclonal antibodies. After washing, the cells were incubated for 1 hour at 37°C with Streptavidin-AP and after another wash, the spots were developed (substrate BCIP-NBT). The number of spots per well was counted automatically (ELISpot Reader; Autoimmun Diagnostika, Germany) and the number of spots per 10^6^ cells from individual mice was calculated.

### Statistical analysis

Statistical analysis was performed with dedicated software (GraphPad Prism vs5; GraphPad, USA). For antibody responses, data are presented as mean± SD and analyzed by Student t-test or one-way ANOVA followed by Bonferroni multiple comparison test. The ELISPOT data are expressed as number of spots per 10^6^ cells± SD (n = 10) and analyzed by one-way ANOVA followed by Bonferroni multiple comparison test. IHC and western blot data are presented as mean± SEM and analyzed by two-way ANOVA followed by Bonferroni multiple comparison tests, by un-paired student t-test or by Mann Whitney test. Survival curves are presented as Kaplan-Meier survival plots and analyzed by Mantel-Cox ranking test and Gehan Breslow Wilcoxon test. The significance level was set at p<0.05.

## Results

Previously, we developed and validated a liposome-based vaccine to target amyloid peptides in AD [21]–[23]. Here, we adapted the liposome technology to target a pathological conformer of protein Tau, using a synthetic peptide of 16 amino acids, that corresponds to a presumed pathological Tau epitope: Tau393-408[pS396/pS404].

**Figure 1:**
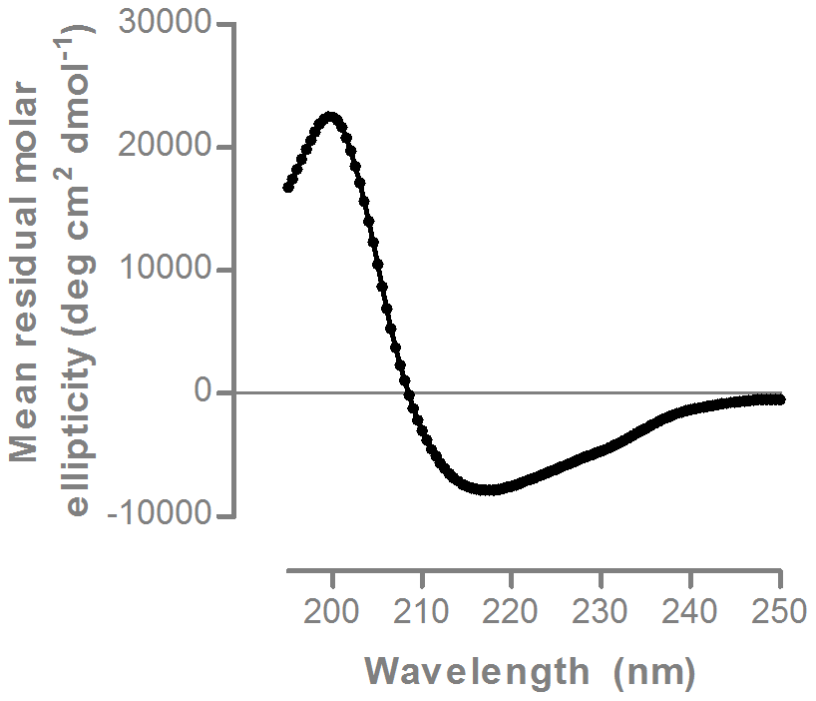
CD spectrum of ACI-35 corresponds to β-sheet secondary structure. The CD spectrum of ACI-35 at (1∶9) dilution in PBS. The spectrum of liposomes lacking the phospho-peptide was subtracted to the signal of ACI-35 for baseline correction. The spectrum shows a maximum around 199.5 nm and a broad minimum around 218 nm.

The peptide was prepared by solid-phase peptide synthesis, with incorporation of phosphorylated serine amino acids at the defined positions S396 and S404. The phospho-peptides were derivatized with two palmitic acid chains at each terminus to enable integration into liposomes. Thereby presentation of the peptides in specific conformations is possible on the liposome surface as demonstrated for the amyloid-based liposomal vaccine [22]–[24]. CD spectrometry of the ACI-35 liposomal vaccine demonstrated that the incorporated phospho-Tau peptides present in an ordered structure. The CD spectra showed a maximum at 199.5 nm and a broad minimum around 218 nm (Figure 1), defining a β-sheet configuration of the Tau393-408[pS396/pS404] peptides on the liposomal surface similar to that of aggregated Tau.

### Immune response against protein Tau in wild-type and Tau.P301L mice

We administered the Tau-liposomal preparation as vaccine first to wild-type mice, and then to transgenic Tau.P301L mice that are pre-clinical models for neuronal tauopathy [12], [30]–[38]. Not only did we assess the potency of the vaccine in generating antibody responses, but more importantly we analyzed the safety aspects of this vaccine that is aimed at a self-antigen in the Tau.P301L mice. Although protein Tau is located intracellularly in central neurons, bound to microtubules, it is also secreted into the interneuronal space and ends up in cerebrospinal and lumbar fluids.

The liposome-based vaccine elicited, already after two sub-cutaneous injections, robust antibody responses in wild-type mice, as well as in Tau.P301L mice. The titers and specificity of the responses were evaluated by ELISA on synthetic peptides and recombinant protein Tau. In addition, the antisera were evaluated by TAUPIR on sections from old biGT mice that develop massive forebrain tauopathy with ageing [31].

**Figure 2:**
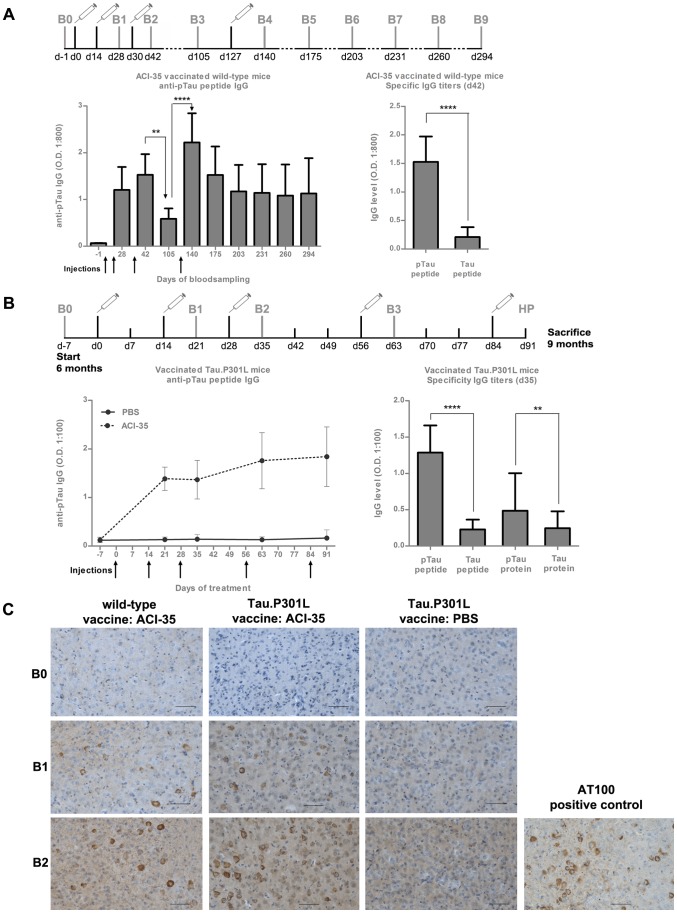
ACI-35 elicits robust and specific antisera against Tau in wild-type and Tau.P301L mice. (A) Vaccination schedule with ACI-35 in wild-type mice is shown schematically with s.c. injections represented by the syringes and the bleedings by the letter B with a number. Antisera titers were measured by ELISA on the phosphorylated antigenic sequence incorporated in the vaccine (pTau peptide), and on the non-phosphorylated peptide of the same primary amino acid sequence (Tau peptide) (see text for details). Data are presented as mean± SD. Statistical analysis: one-way ANOVA followed by Bonferroni multiple comparison test (**p<0.01, **** p<0.0001) and by unpaired student's t-test (**** p<0.0001). (B) Similar vaccination with ACI-35 of Tau.P301L mice and analysis by ELISA. Data are presented as mean± SD. Statistical analysis by unpaired student's t-test (** p<0.01; **** p<0.0001) (C) TAUPIR with antisera from ACI-35 vaccinated wild-type mice and Tau.P301L mice demonstrated a specific reaction with neurofibrillary tangles and neuropil threads in forebrain of biGT mice. IHC with Mab AT100 is included for comparison. IHC with sera from Tau.P301L mice injected with PBS or from Tau.P301L mice that were not vaccinated, were devoid of specific antibodies and auto-antibodies against human protein Tau. Scale bars: 50 µm.

The IgG antibodies produced in wild-type mice after two s.c. injections were specific for the phosphorylated Tau peptide (pTau peptide) in ELISA. Reaction with the non-phosphorylated synthetic peptide with the same amino acid sequence (Tau peptide) was negative or minimal (Figure 2A). Moreover, in TAUPIR the produced antibodies reacted intensely with neurofibrillary tangles and neuropil threads in brain sections of old biGT mice (Figure 2C).

A third immunization of the same wild-type mice did not further increase pTau specific IgG titers (bleeding B2, day 42, Figure 2A), while subsequent discontinuation of vaccination significantly reduced the titers (bleeding B3, day 105). Of note, one additional immunization with the same ACI-35 vaccine reinstated the original high antiserum titers (bleeding B4, day 140), which moreover remained high for at least 5 months without further vaccinations (Figure 2A).

The initial studies in wild-type mice established that the ACI-35 liposome-based vaccine mimicking phosphorylated protein Tau, induced high- and phospho-specific IgG responses already after 2 s.c. immunizations and that two additional inoculations led to sustained high titers for longer periods of time.

Comparably, a regime of bi-weekly administrations of the same vaccine was imposed in transgenic Tau.P301L mice, age 6 months at the first injection. At this age, Tau.P301L mice are cognitively impaired, but still pre-symptomatic in terms of the severe, moribund clinical phenotype that they develop later in life, comprising motor problems, loss in bodyweight and upper-airway defects [30]–[38].

Very similar to the outcome in wild-type mice, two initial s.c. immunizations of transgenic Tau.P301L mice generated high IgG titers specific for the pTau peptide in ELISA (Figure 2B). The antisera also reacted specifically with recombinant Tau protein isolated from transfected yeast cultures [39], [40] (Figure 2B). Negative to weak reaction was observed in ELISA against the non-phosphorylated Tau peptide and against recombinant non-phosphorylated Tau protein produced in bacteria. Serum from naive or control vaccinated Tau.P301L mice were devoid of immunoglobulins that reacted with phosphorylated or non-phosphorylated Tau peptides or with the full-length recombinant Tau proteins (Figure 2B).

The overall outcome demonstrated that the novel Tau-based liposomal preparation was a potent vaccine that elicited high-titered, specific antisera against phosphorylated protein Tau, both in wild-type and in Tau.P301L mice. Interestingly, the Tau.P301L mice were hereby also demonstrated not to produce auto-antibodies against the transgenic human Tau.P301L protein (Figure 2C).

### Efficacy: Brain pathology of vaccinated Tau.P301L mice

Mutant human Tau in Tau.P301L mice becomes phosphorylated at various physiological and pathological epitopes, e.g. pS199, pS202, pT205, pT231, pS262, pS396, pS400, pS404 and pS422 [30], [31]. Tau pathology that progressively develops in ageing Tau.P301L mice, comprises typical fibrillar deposits in soma and neuropil threads, directly visualized by IHC with AT100 and similar antibodies. Closely correlated with the appearance of fibrillar Tau aggregates in neuronal somata and processes, is the biochemical detection of sarcosyl-insoluble Tau, denoted as SInT, which appears first around age 6–7 months in hindbrain and later, around age 9 months, in forebrain of the Tau.P301L mice [30]–[38].

**Figure 3:**
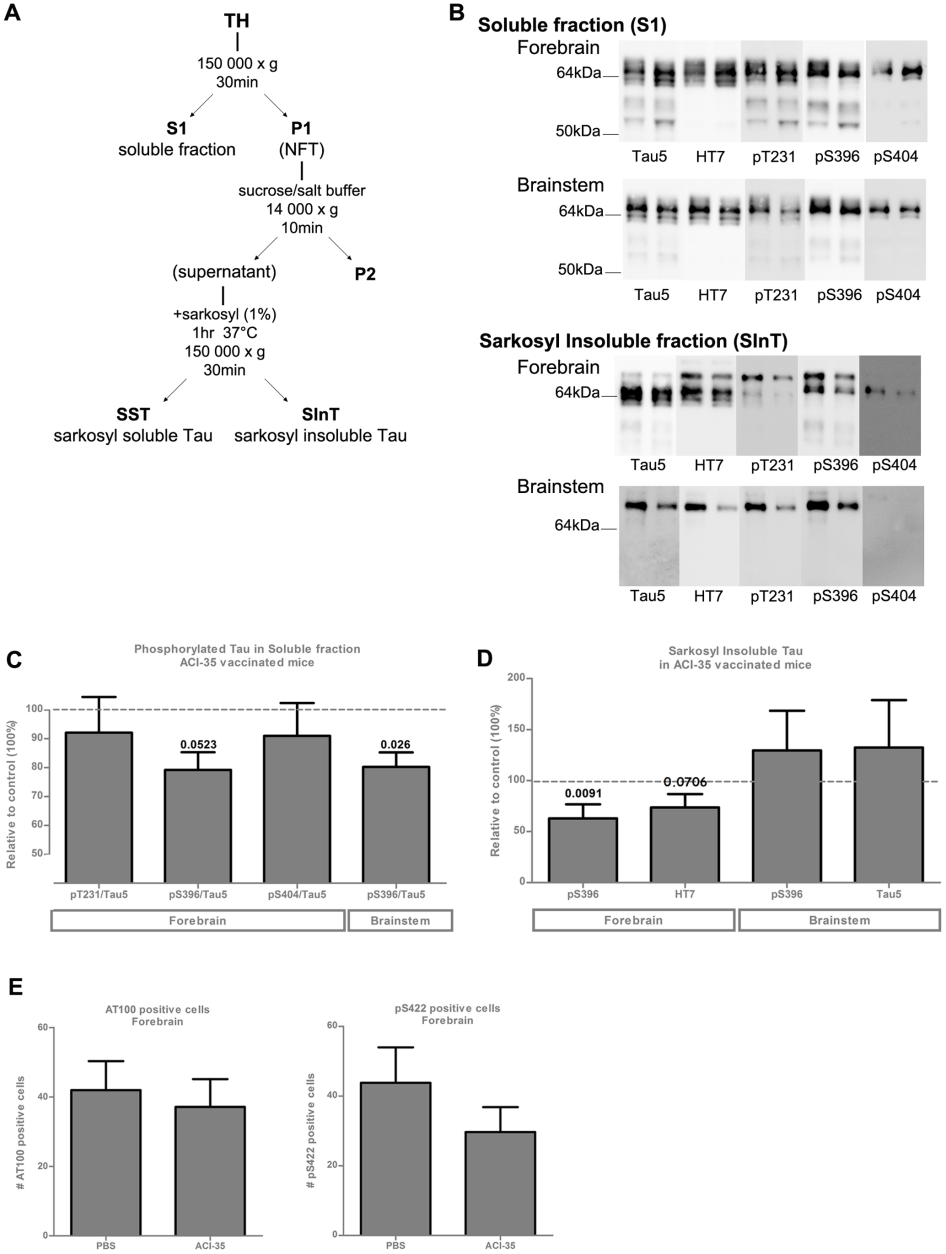
Biochemical analysis of brain from Tau.P301L mice vaccinated with ACI-35. (A) Fractionation scheme of total brain homogenates from Tau.P301L mice to generate soluble (S1) and sarcosyl insoluble fractions (SInT). (B) Representative western blots of S1 and SInT fractions from forebrain and from brainstem of two untreated terminal Tau.P301L mice (age 10 months) developed for total protein Tau (Mab Tau5), for total human protein Tau (Mab HT7) and for phosphorylated Tau (antibodies pT231, pS396 and pS404 as indicated). (C) Reduction of pS396 in soluble fraction of brainstem and forebrain (p = 0.026 and 0.0523, respectively, Student's t-test) from ACI-35 vaccinated Tau.P301L mice, relative to PBS injected mice. (D) Reduction of pS396 (p = 0.0091, Mann Whitney test) and HT7 (p = 0.0706, Mann Whitney test) in SInT in forebrain by ACI-35 vaccination of Tau.P301L mice. (E) Reduction of tangled neurons, marked by IHC for AT100 or pS422, in the forebrain of ACI-35 vaccinated Tau.P301L mice after 3 months of treatment. Data: mean± SEM.

To evaluate biochemically the effects of vaccination with ACI-35, we measured total levels of protein Tau as well as selected phosphorylated epitopes in the soluble (S1) and SInT fractions isolated from forebrain and brainstem of ACI-35 vaccinated Tau.P301L mice (n = 35), compared to control vaccinated Tau.P301L mice (n = 33) (Figure 3A,B). Western blotting with specific phospho-Tau monoclonal and polyclonal antibodies revealed the significant reduction of the pS396 epitope in the soluble fraction of brainstem (p = 0.028), and to a lesser extent in forebrain (p = 0.052) in ACI-35 vaccinated, relative to placebo treated Tau.P301L mice (Figure 3C). However, the levels of protein Tau phosphorylated at residues S404 and T231 were not markedly affected by ACI-35 vaccination. Moreover, insoluble Tau phosphorylated at S396 was also significantly decreased in the forebrain, resulting in decreased total SInT levels quantified with HT7 (Figure 3D). The biochemical findings were corroborated by IHC, showing trends for decreased numbers of AT100 and pS422 positive neurons in the forebrain of vaccinated Tau.P301L mice (Figure 3E).

The difference between biochemical and IHC data was attributed to the fact that IHC did not include neuropil threads, which represent the major fraction of the total tauopathy that is quantified biochemically as the SInT fraction.

We concluded that the levels of specific IgG induced by the ACI-35 vaccine decreased the levels of soluble phosphorylated Tau, as well as the levels of insoluble aggregated Tau in the brain of vaccinated Tau.P301L mice.

### Efficacy: Clinical parameters of vaccinated Tau.P301L mice

Ageing Tau.P301L mice from 7–8 months onwards suffer progressively motor defects, visualized directly by clasping as index of paralysis of hind- and forelimbs, and observed in most Tau transgenic mice. In Tau.P301L mice, the motor problems eventually result in muscle wasting and loss of bodyweight, paralleling the progressive development of tauopathy especially in the hindbrain [30], [31]. Overall, the clinical phenotype develops over a period of about 3 months with variable age of onset in individual mice, but leading invariably to precocious death of Tau.P301L mice, mostly between age 8 to 11 months. Median mortality was at 9.5 months without gender differences and without survivors beyond age 12 months. The terminal stage before death evolved over a period of 2 to 4 weeks after the first signs of paralysis appear and is characterized by severe upper-airway defects, resulting from brainstem tauopathy [30]–[38].

**Figure 4:**
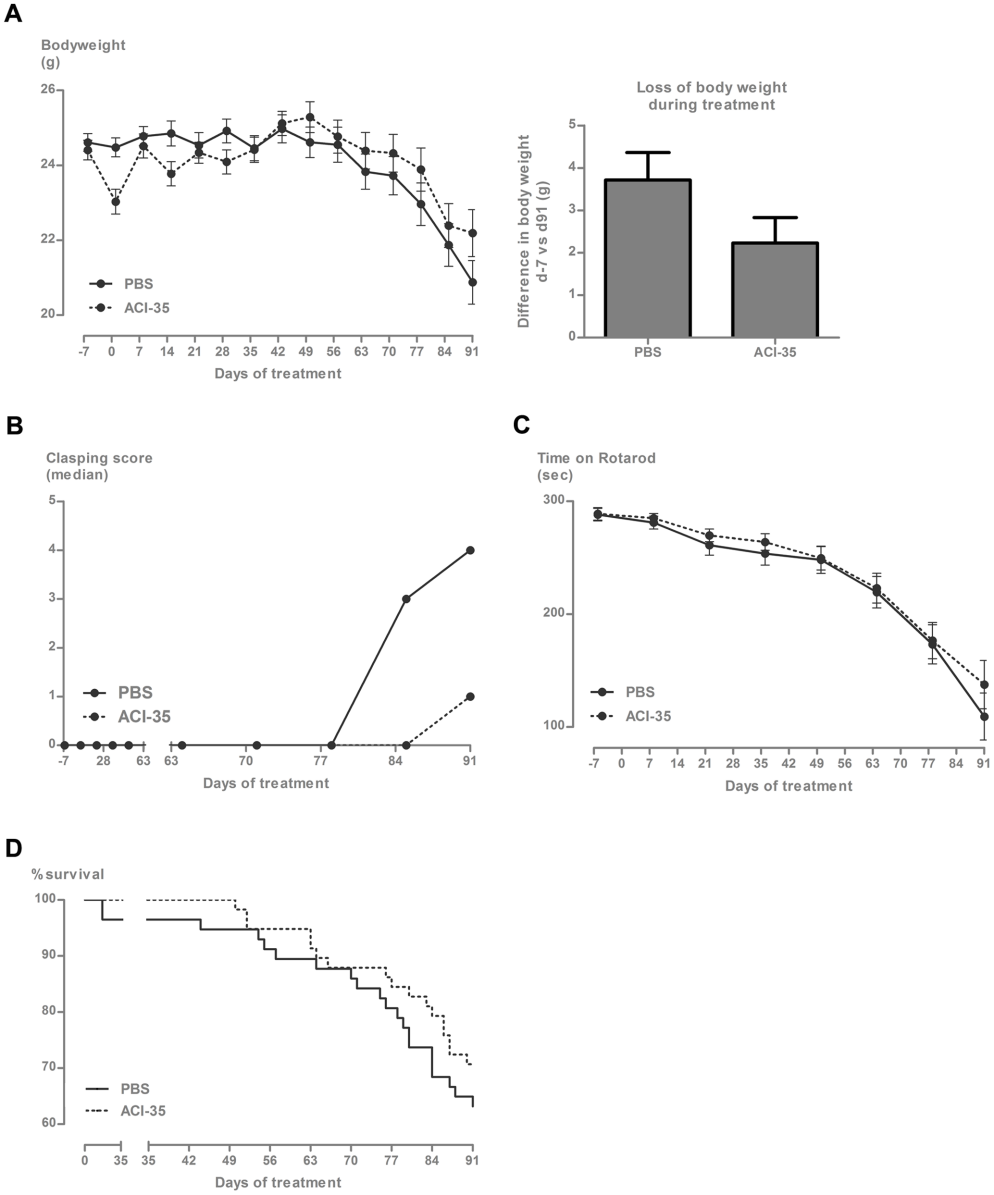
Improved clinical parameters of Tau.P301L mice vaccinated with ACI-35. (A) The left panel shows the evolution of the bodyweight of Tau.P301L mice over 3 months of ACI-35 vaccination relative to PBS injected Tau.P301L mice. The panel on the right presents the loss in bodyweight of the Tau.P301L mice at day 91 relative to the start of the study (day -7). Data: mean± SEM (p = 0.094, unpaired Student's t-test). (B) Clasping score of vaccinated Tau.P301L mice compared to placebo injected Tau.P301L mice. (C) Rotarod performance of ACI-35 vaccinated Tau.P301L mice compared to PBS-injected Tau.P301L mice, tested over 300 sec on the accelerating rod (see Methods section for details). Data: mean± SEM. (E) Mortality of Tau.P301L mice vaccinated with ACI-35 versus placebo Tau.P301L mice.

In the current study, the clinical parameters of clasping and loss of bodyweight were monitored weekly, while motor performance was measured every 2 weeks by the accelerating rotarod test. Vaccination with the experimental liposome-based Tau vaccine ACI-35 did not exert any additional negative effects on any of these parameters. In contrast, the vaccinated mice maintained their bodyweight better than non-vaccinated and PBS-injected Tau.P301L mice (Figure 4A). We observed a minor decrease in bodyweight 24 hr after the first and second vaccinations, likely caused by the acute phase reaction. However, bodyweight became completely normalized 48 hr after the vaccination, while the negative effect became less evident upon subsequent vaccinations of the same mice.

Clasping, as the most direct indicator of motor impairment, was also positively affected by ACI-35 immunotherapy. In vaccinated Tau.P301L mice, clasping was delayed and at the end of the observation period, fewer ACI-35 vaccinated mice showed signs of clasping compared to placebo treated Tau.P301L mice (Figure 4B). Nevertheless, the performance of the vaccinated Tau.P301L mice on the accelerating rotarod was similar to that of control Tau.P301L mice: all mice deteriorated gradually and similarly, which we attributed to the inherently more demanding nature of the accelerating rotarod task (Figure 4C). Finally, ACI-35 vaccinated Tau.P301L mice survived longer than non-vaccinated or PBS treated Tau.P301L mice (Figure 4D).

Overall, the combination of the four clinical indices demonstrated that the liposomal Tau-based vaccine was safe as demonstrated by the absence of adverse effects, the lower clasping score, the attenuated loss of bodyweight and the prolonged survival of ageing Tau.P301L mice. Moreover, the improved clinical parameters in Tau.P301L mice supported the biochemical and immunohistochemical parameters: decreased levels of insoluble protein Tau and less tangled neurons in the brain of vaccinated Tau.P301L mice.

### Safety of liposome-based Tau vaccination

Clinical trials of vaccines that target self-antigens in the CNS have encountered serious safety problems and had to be abrogated because of severe side-effects in patients. These clinical findings emphasize the importance of early consideration of safety aspects, already in preclinical studies to define and anticipate unwanted reactions triggered by the designed vaccine. We therefore analyzed several important indices of inflammation in the brain of vaccinated Tau.P301L mice: the levels of immunoglobulins in brain parenchyma, the activation of astrocytes and microglia, and the extravasation of CD45 positive T and B cells.

**Figure 5:**
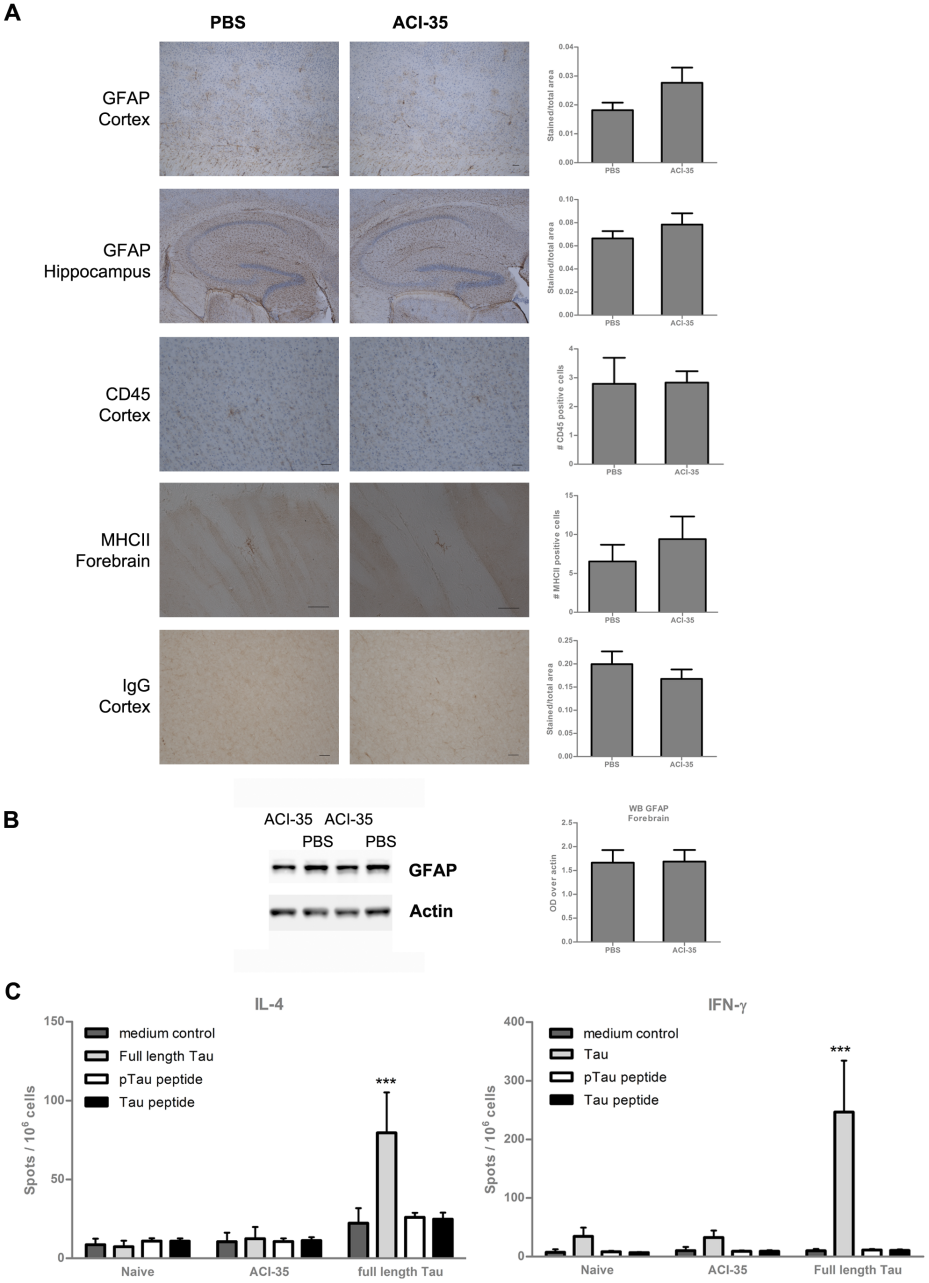
No increased inflammatory response in ACI-35 treated Tau.P301L mice. (A) IHC did not reveal marked differences in inflammation-related parameters in forebrain of ACI-35 vaccinated Tau.P301L mice, relative to PBS-injected Tau.P301L mice. IHC reaction with the different specific antibodies, specified in the captions, was analyzed by image analysis (details see Methods section) and presented as mean± SEM. Scale bars: 50 µm. (B) Western blotting for GFAP of total brain homogenates from Tau.P301L mice vaccinated with ACI-35 (n = 34) or injected with PBS (n = 33). Data presented as mean± SEM. (C) ELISPOT analysis of IFN-γ and IL-4 production by T cells isolated from spleens of naive mice or from mice immunized by either ACI-35 or with recombinant protein Tau. Splenocytes were re-stimulated with medium (cells alone), recombinant Tau protein (100 µg/ml) or with the phosphorylated peptide used in the ACI-35 vaccine and with its un-phosphorylated counterpart (1 µg/ml). Results are expressed as the number of foci (spots per million cells) +SD (n = 10 mice). Statistical analysis: two-way ANOVA followed by Bonferroni multiple comparison test (*** p<0.001).

IHC for GFAP, as marker for astrocytes, revealed no significant induction of astrogliosis in cortex and hippocampus of mice vaccinated with ACI-35 (Figure 5A). The finding was confirmed by western blotting for GFAP in total brain homogenates (Figure 5B). Furthermore, IHC also failed to show microglial activation, or increased immunoglobulin levels, while the number of CD45-positive cells was not increased following Tau-based liposomal vaccination of Tau.P301L mice (Figure 5A). The Tau.P301L mice do not develop marked neuroinflammation with ageing, and the absence of inflammatory markers in the vaccinated mice therefore consolidated the conclusion that no extra inflammatory burden was imposed by the Tau-based liposomal vaccine.

A more elaborate analysis of eventual T cell responses induced by the vaccine was performed by ELISPOT (Figure 5C) because amyloid targeted immunotherapy has caused over-reactive T cell responses with severe side-effects. As described in the previous section, immunization of wild-type mice with ACI-35 induced the production of antisera that were specific for phosphorylated protein Tau. We also immunized wild-type mice with a vaccine containing recombinant protein Tau together with adjuvants Alum and CpG, which produced antisera that reacted with recombinant protein Tau (data not shown). In ELISPOT, re-stimulation with full-length Tau of splenocytes isolated from mice immunized with the Tau/Alum/CpG vaccine induced the production of both IFN-γ and IL-4. This contrasted with splenocytes isolated from ACI-35 immunized mice that were challenged with either full-length Tau protein, pTau peptide or non-phosphorylated Tau peptide: none of these induced production of either IFN-γ or IL-4 (Figure 5C).

We concluded that baseline levels of the different inflammation-related parameters were maintained in the brain of vaccinated Tau.P301L mice. This was confirmed by independent ex vivo tests, demonstrating that no marked inflammation was caused by the Tau-liposomal vaccine in Tau.P301L mice.

## Discussion

Previously, the liposomal vaccine that targets amyloid peptides was proven to be safe and efficacious in preclinical studies and is now evaluated in clinical settings for immunotherapy of Alzheimer's disease [21]–[24]. Here we present the development and characterization of a liposome-based vaccine aimed at a prominent phosphorylated epitope of protein Tau that is pathologically important.

### Specificity & Efficacy

The selected antigen was a 16-mer synthetic peptide, corresponding to sequence 393–408 of protein Tau and phosphorylated at residues S396 and S404. The peptides were anchored into the liposomal bilayer via unique N- and C-terminal palmitoylated lysine residues. We demonstrated for the amyloid vaccine that the peptides adopt a structured conformation on the liposomal surface [23]. The CD spectrum analysis reported here, corroborates the conclusion that similar to amyloid peptides, the phosphorylated Tau393-408 peptide can adopt a β-sheet conformation on the liposomal surface. Moreover, the crisp visualization of the typical tauopathy in brain of biGT mice confirms the induction of sequence and/or conformation specific antibodies in the polyclonal response against the ACI-35 vaccine.

The combined elements and composition of the ACI-35 vaccine proved to be strongly immunogenic in wild-type mice, as well as in Tau.P301L transgenic mice: stable, high-titer polyclonal antibody responses were elicited already after two sub-cutaneous vaccinations. Moreover, ELISA demonstrated that the polyclonal responses were specific for phosphorylated protein Tau.

The generation of phosphorylation and conformation specific antisera by vaccination with ACI-35 in Tau.P301L mice corroborated the reduction of protein Tau phosphorylated at S396 in the soluble fraction of forebrain and brainstem. In contrast, levels of pS404-Tau and pT231-Tau were only minimally affected, indicating that the vaccine preferentially acted on pS396-Tau isoforms. A similar trend was evident in the sarcosyl insoluble Tau fraction of the forebrain of vaccinated Tau.P301L mice.

### Safety

Besides efficacy in generating antigen-specific immune responses, it is important to consider safety aspects with respect to the type of antibodies generated. Unexpected autoimmune reactions and secondary effects need to be taken into account in the development of targeted immunotherapy against protein Tau [41]. Antigens that carry many T-cell epitopes are more likely to elicit aberrant CNS inflammation as observed in clinical and preclinical studies with amyloid peptides [19], [20], [42]–[44]. The ACI-35 vaccine combines the repetitive display of the antigenic peptide on the liposomal surface with the presence of MPLA as adjuvant. MPLA is a non-toxic version of lipid A, well-known to promote immunochemical reactions in vivo by boosting immunoglobulin production in a T-cell independent manner. The likely mechanism involves TLR4 and TRIF-signaling on B-lymphocytes, obviating negative effects of other forms of lipid A that can induce additional inflammatory responses [24], [45], [46].

The ELISPOT data included in the current study confirmed that also the liposomal Tau vaccine induced the generation of specific antibodies without detectable T-cell responses. The outcome was corroborated by the total lack of inflammation in the brain of vaccinated wild-type or Tau.P301L mice, and by the general improvement of the clinical condition of the vaccinated Tau.P301L mice.

The reported data endorse the safety profile of this type of vaccine, even after multiple and prolonged administration. Moreover, the composition of the liposomes obviates the need for extra adjuvants, which in AD patients have shown to cause considerable side-effects [19], [20], [42]–[44]. Finally, derivatives of lipid A are approved for clinical treatment in humans, which facilitates the eventual transition from preclinical studies to clinical trials.

The experimental demonstration that the ACI-35 vaccine was immunogenically most specific for pS396-Tau and improved the clinical condition of Tau.P301L mice, endorses the hypothesis that liposomal vaccines against dominant pathological epitopes are both safe and effective. Additional liposome-based vaccines can now be developed to target other pathological phosphorylated epitopes, to increase the clearance of pathological Tau isoforms and the positive effect on the clinical condition. Moreover, liposomal vaccines targeting protein Tau can be combined with those developed previously against amyloid peptides to eventually provide active immunotherapy that targets concurrently both pathological protein conformers in the brain of Alzheimer's patients.

### Mode of action

The confirmation with a novel type of vaccine that immunotherapy against protein Tau is an interesting therapeutic strategy, raises basic questions regarding the mechanism of action. In general, all studies of immunotherapy for neurodegenerative diseases require more extensive elaboration, and we can only speculate how the generated polyclonal immune response might act on protein Tau in the brain of Tau.P301L mice.

Whether the antibodies act in the circulation or cross the blood-brain-barrier and exert effects locally in the brain parenchyma, remains an open question. Only a small fraction of circulating immunoglobulins enters the brain tissue in healthy individuals, although exact data still encounter considerable analytical problems. Immunoglobulins can cross the BBB by transcytosis, but also infiltration of plasma cells and local production of antibodies has been proposed [47]–[53]. Moreover, in AD and other neurological disorders, the BBB is compromised which might allow antibodies to enter the CNS more readily [47]–[53].

Other experimental findings maintain that immunoglobulins might not have to cross the BBB to be effective. The ‘sink effect’ states that immunotherapy of CNS targets, such as amyloid, can be explained by antibodies in the circulation that capture antigens in the blood and thereby enhance their elimination from the CNS [54]. This hypothesis was also postulated for immunotherapy of protein Tau [55], but we failed to detect circulating immune complexes containing protein Tau.

Protein Tau is a large, naturally unfolded, intracellular protein which poses extra problems compared to the small extracellular amyloid peptides. Several preclinical studies of immunotherapy targeting protein Tau have now been published [55]–[62]. Moreover, other intracellular proteins have been positively evaluated as targets for immunotherapy: alpha-synuclein in a mouse model for Parkinson's disease [63] and superoxide dismutase in an ALS mouse model [64]. The combined data add weight and growing belief in the feasibility of immunotherapy against intracellular proteins, and protein Tau in particular.

Conversely, the intracellular fibrils of aggregated protein Tau are, on a molecular scale, enormous in size and moreover not readily reversible. In addition, the large fibrils are not the prime neurotoxic species in tauopathy, which combined with stoichiometric problems, eliminates them as primary targets of immunotherapy. Experimental evidence corroborates the hypothesis that the cause of neuronal, and thereby of cognitive dysfunction, are smaller oligomeric aggregates of as yet not clearly defined size and molecular constellation [12], [33], [65]–[68]. Moreover, suggestions that tauopathy can spread among neurons by intercellular transfer of protein Tau, phosphorylated or not, also explains the presence of protein Tau in interstitial, cerebrospinal and lumbar fluids [69]. Nevertheless, whereas brain-regional spreading of tauopathy must by definition be restricted to pathology, the fact that protein Tau is also present in CSF of healthy, normo-cognitive persons remains unexplained. Taking this one step further, means that protein Tau can escape from neurons either by accidental leakage, or on purpose to serve a physiological function in signal transmission or another as yet unknown feature.

Without answering these important physiological questions, we can still envisage that in pathology the undefined normal mechanism can serve as template, or is hijacked by a non-physiological mechanism. The non-conventional release of protein Tau then explains (i) its presence in CSF, even in the absence of neuronal damage [69]; (ii) its eventual contribution to the regional propagation of tauopathy [70]–[72]; and (iii) the beneficial effects of Tau-directed vaccination, which then acts not on an intracellular, but on an extracellular target. Obviously, the beneficial effect of immunotherapy as a consequence of proteins being targeted extraneuronally is the more acceptable, although still not fully understood mode of action in brain.
